# Green tea extract enhances parieto-frontal connectivity during working memory processing

**DOI:** 10.1007/s00213-014-3526-1

**Published:** 2014-03-19

**Authors:** André Schmidt, Felix Hammann, Bettina Wölnerhanssen, Anne Christin Meyer-Gerspach, Jürgen Drewe, Christoph Beglinger, Stefan Borgwardt

**Affiliations:** 1 Department of Psychiatry (UPK), University of Basel, Wilhelm Klein Str. 27, 4012 Basel, Switzerland; 2 Medical Image Analysis Center, Schanzenstrasse 55, 4031 Basel, Switzerland; 3 Department of Gastroenterology, University Hospital Basel, 4031 Basel, Switzerland; 4 Department of Psychosis Studies, Institute of Psychiatry, King’s College London, London, UK

**Keywords:** Cognition, Working memory, Green tea extract, Brain activity, Effective connectivity, Dynamic causal modeling

## Abstract

**Rationale:**

It has been proposed that green tea extract may have a beneficial impact on cognitive functioning, suggesting promising clinical implications. However, the neural mechanisms underlying this putative cognitive enhancing effect of green tea extract still remain unknown.

**Objectives:**

This study investigates whether the intake of green tea extract modulates effective brain connectivity during working memory processing and whether connectivity parameters are related to task performance.

**Material and methods:**

Using a double-blind, counterbalanced, within-subject design, 12 healthy volunteers received a milk whey-based soft drink containing 27.5 g of green tea extract or a milk whey-based soft drink without green tea as control substance while undergoing functional magnetic resonance imaging. Working memory effect on effective connectivity between frontal and parietal brain regions was evaluated using dynamic causal modeling.

**Results:**

Green tea extract increased the working memory induced modulation of connectivity from the right superior parietal lobule to the middle frontal gyrus. Notably, the magnitude of green tea induced increase in parieto-frontal connectivity positively correlated with improvement in task performance.

**Conclusions:**

Our findings provide first evidence for the putative beneficial effect of green tea on cognitive functioning, in particular, on working memory processing at the neural system level by suggesting changes in short-term plasticity of parieto-frontal brain connections. Modeling effective connectivity among frontal and parietal brain regions during working memory processing might help to assess the efficacy of green tea for the treatment of cognitive impairments in psychiatric disorders such as dementia.

## Introduction

Recent research indicates that green tea extract or its main ingredients has a beneficial impact on cognitive functioning in humans. For instance, it has been demonstrated that the consumption of green tea improved memory and attention in subjects with mild cognitive impairments (Park et al. 2011) and that the consumption of flavonoid-rich foods such as green tea reduced beta-amyloid-mediated cognitive impairments in Alzheimer transgenic mice, suggesting a potential therapeutic utility in dementia (Rezai-Zadeh et al. 2008; Williams and Spencer 2012). Furthermore, higher consumption of green tea has also been associated with a lower prevalence of cognitive impairments in older adults (Kuriyama et al. 2006). Comparable results were obtained in another study investigating the association between green tea consumption and cognition in 2,501 people aged over 55 years by showing that the intake of green tea was significantly related to a lower prevalence of cognitive impairments (Ng et al. 2008). In addition to preventing cognitive decline, green tea consumption might even lead to better cognitive performances in community-living older adults (Feng et al. 2010), which may indicate a cognitive enhancing effect in healthy subjects.

More recently, a study used functional magnetic resonance imaging (fMRI) to investigate whether this beneficial impact of green tea on cognition could be related to altered brain activity in regions crucially engaged during higher-order cognitive functioning (Borgwardt et al. 2012). They demonstrated relatively increased brain activation in fronto-parietal regions, most pronounced in the right frontal cortex after the administration of green tea extract during working memory (WM) processing as assessed by the N-back task (Borgwardt et al. 2012). These data suggest that green tea extract may modulate brain activity in key areas for mediating WM processing in the human brain such as the dorsolateral prefrontal cortex (Goldman-Rakic 1996). However, successful WM processing during the N-back task requires a functional coupling of parietal and frontal brain regions as shown by functional (Owen et al. 2005; Rottschy et al. 2012) and effective connectivity studies (Deserno et al. 2012; Ma et al. 2011). It has been suggested that effective connectivity from the parietal cortex to the frontal cortex may contribute to the encoding of incoming stimuli (Ma et al. 2011), while the connections from the frontal to the parietal cortex likely mediate the updating of rules (e.g., 2-back condition; Gazzaley et al. 2004; Sauseng et al. 2005). Therefore, it is conceivable that the increased frontal activity during WM processing after green tea administration (Borgwardt et al. 2012) may have resulted from a change in functional coupling connectivity from the parietal to the frontal cortex.

We thus explored in the current study whether the administration of green tea extract changed brain connectivity between the frontal and parietal cortex during WM processing. In particular, we applied dynamic causal modeling (DCM; Friston et al. 2003) to fMRI data from 12 healthy subjects receiving green tea extract and a control beverage while performing a N-back WM task. DCM can explicitly evaluate the directional modulation effects of contextual experimental conditions (e.g. the 2-back condition) on effective connectivity and has been successfully used to detect pharmacological manipulations from fMRI data (Grefkes et al. 2010; Schmidt et al. 2013b). Furthermore, we tested whether the effect of green tea on the WM-induced modulation of connectivity was related to its effect on the task performance. Given the important functional coupling between parietal and frontal brain regions during the N-back task (Owen et al. 2005; Rottschy et al. 2012), and that the intake of green tea extract increases prefrontal activity (Borgwardt et al. 2012), we hypothesized that green tea extract would enhance effective connectivity from the parietal to the frontal cortex.

## Material and methods

### Participants

In total, 12 healthy right-handed male completed the study (mean age 24.1 years; standard deviation 2.6). All participants were nonsmokers. Participants were told to abstain from any substance use for the duration of the study, and from the intake of alcohol, caffeine, green tea products, and citrus juices for 24 and 12 h before each study day, respectively. At the start of the study, a urine sample was collected for screening for amphetamines, benzodiazepines, cocaine, methamphetamine, opiates, and THC using immunometric assay kits. None of the participants were tested positive on any of the sessions. Participants were carefully screened using a semistructured clinical interview to exclude psychiatric or physical illness or a family history of psychiatric illness. The local State Ethical Committee (Ethikkommission Beider Basel) approved the study and all participants gave their informed written consent after the study procedure had been explained to them in detail. The study was registered with clinicaltrials.gov (identifier: NCT01615289).

### Experimental design

A double-blind, vehicle-controlled, and within-subject design with randomized order of substance administration using an established protocol was conducted over four sessions (Bhattacharyya et al. 2012; Borgwardt et al. 2008). Participants received either 250 or 500 ml milk whey-based soft drink containing 13.75 and 27.5 g of green tea extract, respectively (Rivella, Rothrist, Switzerland), or a milk whey-based soft drink without green tea extract as control condition. Each participant was scanned four times with a 1-week interval between scans. Before each scanning session, participants swallowed a feeding tube for application of the test solutions. The doses of 250 (that were diluted to 500 ml to control for volume effects) and 500 ml were selected to produce an effect on regional brain functioning without provoking any toxic, psychiatric or physical symptoms, which might have confounded interpretation of the fMRI data and caused difficulties for participants to tolerate the procedure. As the intragastric administration bypassed the sensory systems, volunteers were prevented from guessing which treatment they were being given. An intravenous line was inserted in the nondominant arm of each participant at the start of the testing session to monitor substance whole-blood levels. All participants were physically examined before testing and their heart rate and blood pressure were assessed in 5-min intervals throughout the 1-h session.

### Composition of test drinks

Rivella is a commercially available carbonated soft drink on the basis of milk whey. In 1999, a new flavor with a 0.05 % addition of standardized green tea extract was introduced. The control drink is most similar to the drink of interest, apart from the green tea extract, differs primarily in its lower carbohydrate content (2.5 g/100 ml difference). In detail, the test drink contains the following ingredients: water, milk whey 35 %, lactic acid, carbon dioxide, calcium cyclamate, acesulfame K, and the following minerals: sodium 130 mg/l, potassium 450 mg/l, magnesium 35 mg/l, calcium 165 mg/l, and chloride 330 mg/l. Additionally, it contains the following ingredients: green tea extract 0.05 %, ascorbic acid 120 mg/l, pyridoxine 30 mg/l, and fructose 25 g/l. Green tea extract is prepared from the dried green leaves of *Camellia sinensis* with a drug:extract ratio of 5.5:1, 47.5–52.5 % m/m polyphenols [high-pressure liquid chromatography (HPLC)], 5.0–10.0 % m/m caffeine (HPLC), 0.3–1.2 % m/m theobromine (HPLC), and 1.0–3.0 % m/m theanine (HPLC). One gram of extract corresponds to 5.5 g of green tea leaves. To equalize carbohydrate, the control treatments were supplemented with 6.25 or 12.5 g of sucrose for 250 and 500 ml, respectively. To additionally blind volunteers to treatments, 250 ml treatments and controls were diluted to 500 ml with 250 ml of uncarbonated spring mineral water. This preparatory step also ensures equivalent rates of gastric emptying. Treatments were heated to room temperature and freed from carbon dioxide by stirring.

### fMRI paradigm: N-back task

A rapid, mixed trial, event-related fMRI design was used with jittered interstimulus intervals incorporating random event presentation to optimize statistical efficiency (Ettinger et al. 2011). During the N-back task (Broome et al. 2009), all subjects saw series of letters with an interstimulus interval of 2 s. Each stimulus was presented for 1 s. During a baseline (0-back) condition, subjects were required to press the button with the right hand when the letter „X” appeared. During 1-back and 2-back conditions, participants were instructed to press the button if the currently presented letter was the same as that presented 1 (1-back condition) or 2 trials beforehand (2-back condition). The three conditions were presented in ten alternating 30 s blocks (2 × 1-back, 3 × 2-back and 5 × 0-back) matched for the number of target letters per block (i.e., 2 or 3), in a pseudo-random order.

### Image acquisition and analysis

fMRI was performed on a 3T scanner (Siemens Magnetom Verio, Siemens Healthcare, Erlangen, Germany) using an echo planar sequence with a repetition time of 2.5 s, echo time of 28 ms, matrix 76 × 76, 126 volumes and 38 slices with 0.5 mm interslice gap, providing a resolution of 3 × 3 × 3 mm^3^, and a field of view 228 × 228 mm^2^. We analyzed fMRI data using SPM8 (http://www.fil.ion.ucl.ac.uk/spm/). All volumes were realigned to correct for head movements, mean adjusted by proportional scaling, normalized into standard stereotactic space (Montreal Neurological Institute), and smoothed using a 8 mm full-width at half-maximum Gaussian kernel. We convolved the onset times for each condition (0-back, 1-back, and 2-back) with a canonical haemodynamic response function. Serial correlations were removed using a first-order autoregressive model and a high-pass filter (128 s) was applied to remove low-frequency noise. Six movement parameters were also entered as nuisance covariates to control for movement. We focused our analysis on the 2-back >0-back contrast (main effect of task) to capture the highest possible WM load during the N-back task according to previous N-back fMRI studies (Deserno et al. 2012; Schmidt et al. 2013b).

**Fig. 1:**
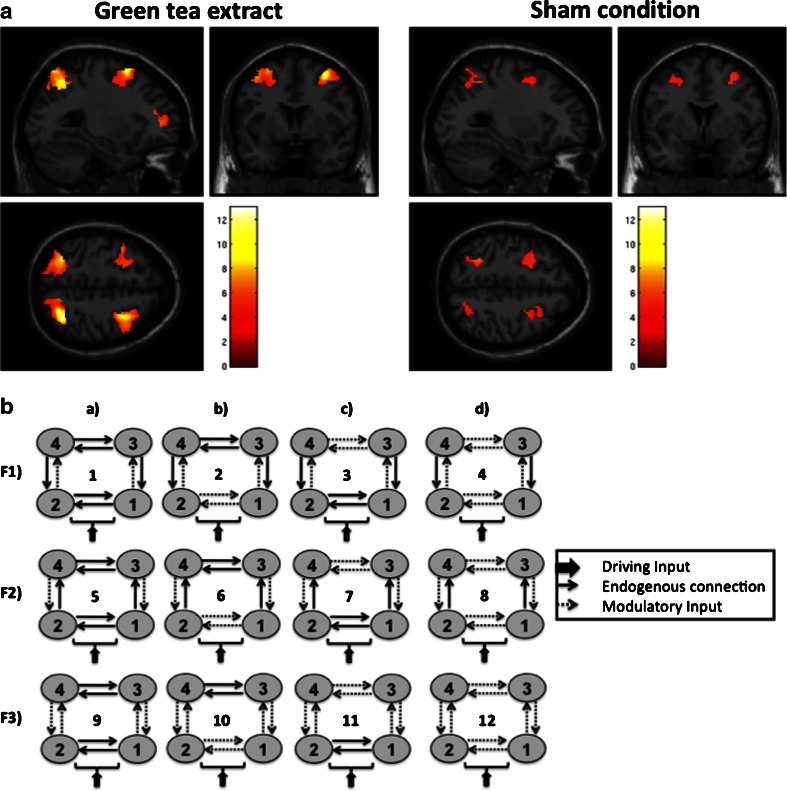
**a** Local maxima with the bilateral superior parietal lobule and middle frontal gyri induced by the main effect of task (2-back >0-back contrast) after the administration of green extract or the of the control substance (FWE cluster level corrected at *p* < 0.05). **b** Model space tested in this study. *1* right SPL, *2* left SPL, *3* right MFG, and *4* left MFG. In particular, we contrasted models in which the 2-back WM condition was allowed to modulate, within both hemispheres: (*F1*) the parieto-frontal connections, (*F2*) the fronto-parietal connections, or (*F3*) both. These three intrahemispheric options were crossed with four possibilities which interhemispheric connections might be modulated by the 2-back WM condition, i.e., (*a*) none (first column of Fig. 1b), (*b*) the interhemispheric connections between parietal areas (second column of Fig. 1b), (*c*) the interhemispheric connections between frontal areas (third column of Fig. 1b), or (*d*) both (fourth column of Fig. 1b). As a result, our model space consisted of 12 alternative models, each of which was fitted to the data from each individual subject

Differences in local brain activity between the different treatment conditions have previously been reported (Borgwardt et al. 2012); here, we extended this study by conducting an effective connectivity analysis using DCM (Friston et al. 2003), which was restricted to the bilateral superior parietal lobule (SPL) and middle frontal gyrus (MFG). As this previous analysis revealed significant differences in fronto-parietal activity especially between the 500 ml doses (Borgwardt et al. 2012), we restricted our connectivity analysis to these two conditions only. The selection of our ROIs were based on the following evidences: (a) the previously published 2-back >0-back contrast of this data (Fig. 1a; Borgwardt et al. 2012), (b) the previous functional connectivity studies emphasizing the importance of fronto-parietal coupling for WM (Gazzaley et al. 2004; Sauseng et al. 2005), and (c) the previous DCM studies of WM (Deserno et al. 2012; Schmidt et al. 2013b). The treatment-specific fronto-parietal network was detected using an anatomical mask taken from the Automated Talairach atlas in the WFU Pick Atlas toolbox (Tzourio-Mazoyer et al. 2002) consisting of the bilateral SPL and MFG. Statistical significance was assessed at the cluster level using the nonstationary random field theory (Hayasaka et al. 2004). The first step of this cluster-level inference strategy consisted of identifying spatially contiguous voxels at a threshold of *p* < 0.001, without correction (cluster-forming threshold; Petersson et al. 1999). Finally, a familywise error (FWE)-corrected cluster-extent threshold of *p* < 0.05 was defined to infer statistical significance.

### Effective connectivity analysis: DCM

DCM10 (revision number 4290) as implemented in SPM8 was used to analyze effective fronto-parietal connectivity during WM processing. In DCM for fMRI, the dynamics of the neural states underlying regional BOLD response are modeled by a bilinear differential equation that describes how the neural states change as a function of endogenous interregional connections, modulatory effects on these connections, and driving inputs (Friston et al. 2003; Stephan et al. 2007). The endogenous connections represent coupling strengths in the absence of inputs to the system (task-independent), while the modulatory effects represent context-specific and additive changes in coupling (task-induced alterations in connectivity). The modeled neuronal dynamics is then related to the measured blood oxygen level-dependent (BOLD) signal using a hemodynamic forward model (Stephan et al. 2007). Here, we explicitly examined how the coupling strengths between frontal and parietal regions are changed by the 2-back condition (modulatory effect).

### Model design and time series extraction

Across all models tested, we assumed the same network layout of connections between right and left SPL and MFG. Specifically, SPL and MFG were reciprocally connected within both hemispheres, with additional interhemispheric connections between all regions. Similar to a recent DCM study of WM (Ma et al. 2011), the visual input (driving) entered the SPL bilaterally (Baizer et al. 1991; Nakashita et al. 2008). Starting from this basic layout, a factorial structured model space was derived by considering where the modulatory effect of the 2-back WM condition might be expressed within both hemispheres (for a graphical summary of the model design see Fig. 1b). Subject-specific regional time series from the SPL and MFG were extracted from spherical volumes of interest with 12 mm in diameter that were centered on the condition maxima of the 2-back >0-back contrast within the anatomical mask taken from the Automated Talairach atlas in the WFU Pick Atlas toolbox (Tzourio-Mazoyer et al. 2002) using the first eigenvariate of voxels above a subject-specific F-threshold of *p* < 0.001 uncorrected. When a subject had no voxel above threshold at the group maxima (Fig. 1a, Table 1), we selected the nearest supra-threshold voxel within the mask. One subject revealed no activated voxels under these criteria and was therefore excluded from the connectivity analysis.

**Table 1:** MNI coordinates (*x*, *y*, *z*) of the treatment maxima during working memory processing

	Left MFG	Right MFG	Left SPL	Right SPL
Green tea extract	(−50, 22, 34) (cluster size: 1199)	(52, 26, 34) (cluster size: 740)	(−30, −62, 48) (cluster size: 895)	(32, −58, 54) (cluster size: 910)
Sham condition	(−36, 4, 64) (cluster size: 220)	(26, 14, 50) (cluster size: 215)	(−34, −54, 56) (cluster size: 255)	(28, −60, 50) (cluster size: 188)

Reported activations survive FWE correction at *p* < 0.05 at peak and cluster level

### Bayesian model selection and Bayesian model averaging

Bayesian model selection (BMS) was used to determine the most plausible neurophysiological network given the data as expressed by a series of competing DCMs. BMS rests on comparing the (log) evidence of a predefined set of models (the model space). The model evidence is the probability of observing the empirical data, given a model, and represents a principled measure of model quality, derived from probability theory (Penny et al. 2004). We used a random-effects BMS approach for group studies, which is capable of quantifying the degree of heterogeneity in a population while being extremely robust to potential outliers (Stephan et al. 2009b). This method considers the model as a random variable and estimates the parameters of a Dirichlet distribution, which describes the probabilities of all models considered. One common way to summarize the results of random effects BMS is to report the exceedance probability (EP) of each model, i.e., the probability that this model is more likely than any other of the models tested, given the group data (Stephan et al. 2009b). Given that different models may be found to be optimal across treatments and statistical comparison of model parameter estimates is only valid if those estimates stem from the same model, Bayesian model averaging (BMA) has been recommended as standard approach for clinical DCM studies (Seghier et al. 2010; Stephan et al. 2010). BMA averages posterior parameter estimates over models, weighted by the posterior model probabilities (Penny et al. 2010). Thus, models with a low posterior probability contribute little to the estimation of the marginal posterior.

### Statistic of DCM parameters

Following BMA, we used the resulting posterior means from the averaged DCM for examining between-treatment differences. In this paper, we focused on WM-induced changes in connectivity. Thus, we tested for group differences in the modulatory effects only. We then used a paired *t* test, testing which of the connectivity parameters differed across the 500 ml treatments.

### Statistics of WM performances

Beyond previous analyses of reaction times and number of errors (Borgwardt et al. 2012), WM performances were objectively quantified using signal detection theory using the formula *d*′ = *z*(Hits)−*z*(FA), where FA reflects false alarms (Macmillan and Creelman 1991). Hit and false alarm rates of zero or one were adjusted as previously described (Macmillan and Kaplan 1985). Paired *t* test was used to assess between-treatment differences in WM performances.

## Results

### Working memory performance

**Fig. 2:**
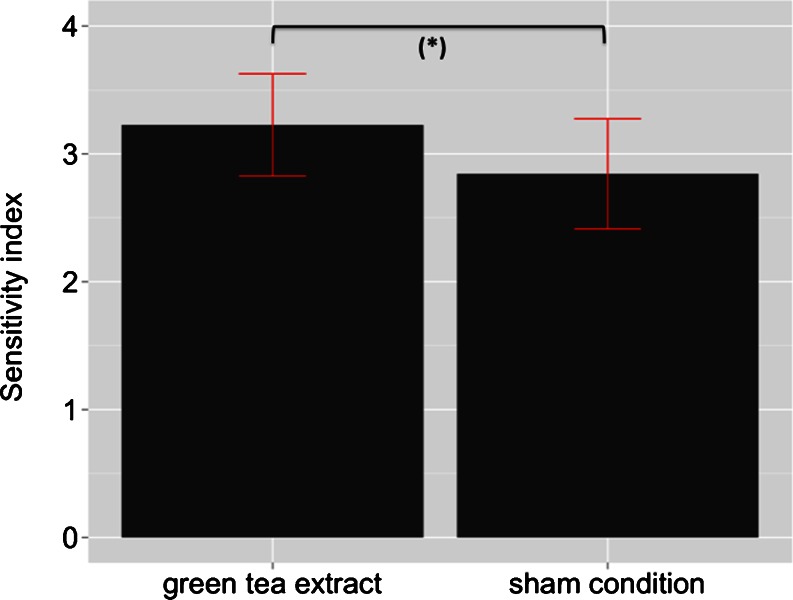
Mean of sensitivity indexes (*d*′) ± SE during working memory processing for both treatment conditions. (*Asterisk*) indicates a between-treatment difference at *p* = 0.066

There was a strong trend toward a significantly improved task performance as expressed by the sensitivity index *d*′ after consumption of green tea extract [mean (SD): 3.23 (0.42)] compared with the control drink [mean (SD): 2.84 (0.45); *t*(11) = 2.041; *p* = 0.066; Fig. 2].

### Bayesian model selection

**Fig. 3:**
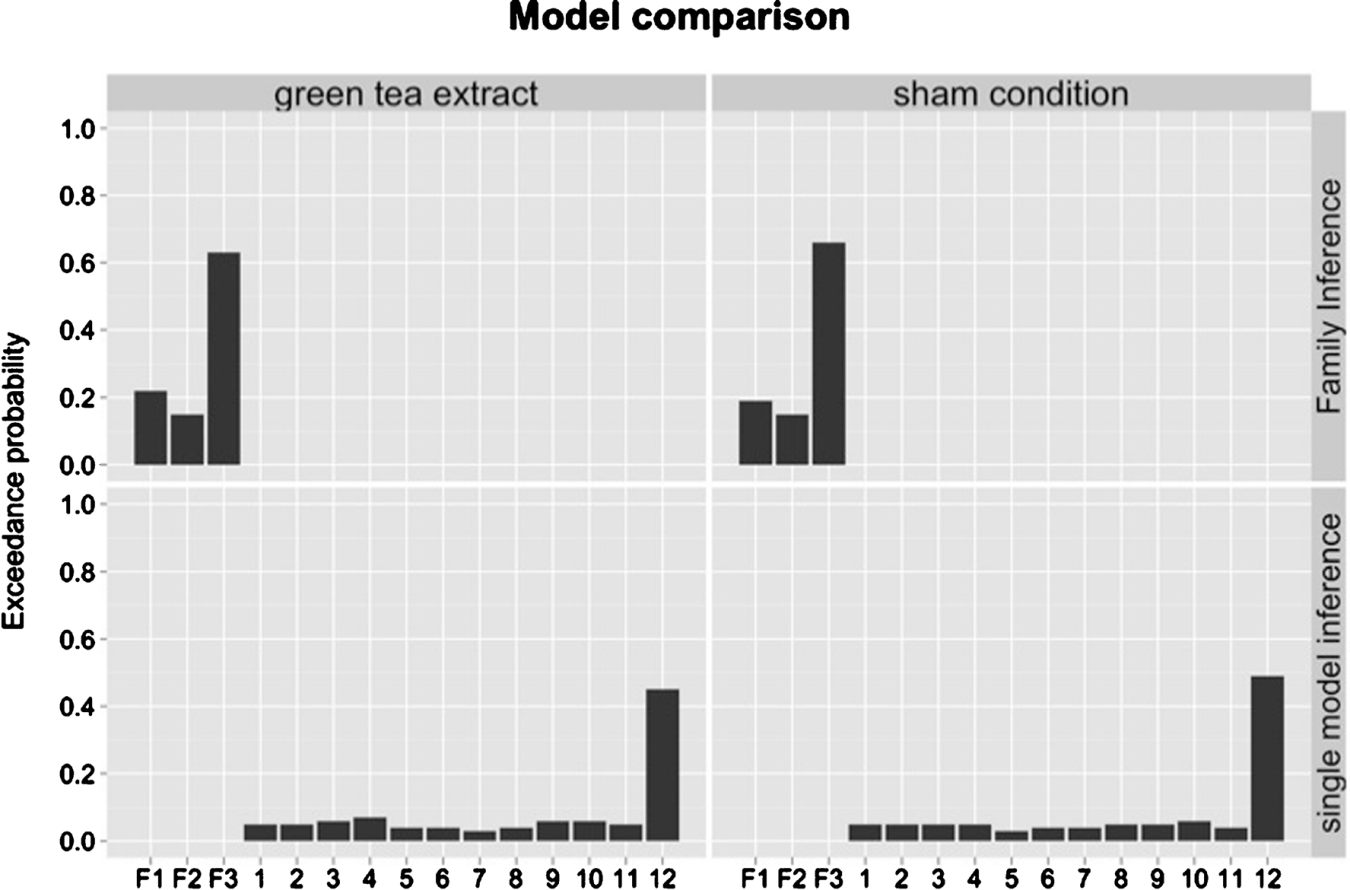
Bayesian model selection (BMS) results on family level (*upper column*) and single model level (*lower column*) over both treatment conditions separately. BMS results are reported in terms of exceedance probabilities

We first used Bayesian model selection (BMS) to compare the model evidence for the three families of models with either bidirectional, forward, or backward modulation of prefrontal–parietal connections. BMS revealed that the family with WM-induced modulation of both forward and backward modulation of prefrontal–parietal connections (F1) was superior to the other families in the green tea (EP 63 %) and control condition (EP 66 %). Single model inference showed that model 12 emerged as the most likely model in the green tea (EP 45 %) and control condition (EP 49 %). These BMS results across both treatment conditions are summarized in Fig. 3.

### Effective connectivity results

**Fig. 4:**
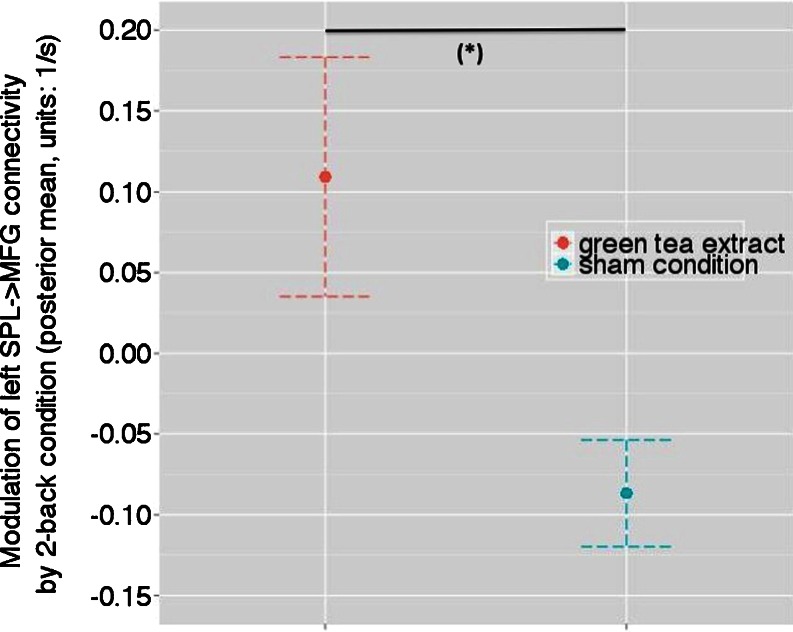
The modulatory effect of the 2-back WM condition on the connection from the right SPL to the right MFG in the sham condition and after the administration of green tea extract. The *y* axis denotes the average over all subjects and all 12 DCMs (using BMA) with regard to the posterior mean (1/s) of the modulatory effect; this encodes changes in connection strength induced by the 2-back WM condition. Significant between-treatment differences at (*asterisk*) *p* < 0.05. Error bars represent standard deviations derived from Bayesian parameter averages

Statistical analysis of treatment differences in connection strengths concerned the posterior means of coupling estimates, following BMA over all 12 models. Thus, in our analysis of effective connectivity, we were able to test for between-treatment differences in eight parameters describing the modulation of fronto-parietal connections, within and across hemispheres, induced by the 2-back WM condition. Paired *t* test results for all connection are summarized in Table 2. A significantly increased WM-induced modulation of connectivity from the right SPL to the right MFG was found in the green tea condition compared with the control beverage [*t*(10) = 2.53; *p* = 0.030; not corrected for multiple comparisons; Fig. 4].

**Table 2:** Paired *t* test results for the between-treatment comparison of connectivity estimates (modulatory effects of 2-back WM condition)

	Paired differences mean	Standard deviation	Standard error mean	*t* Value	Significance (two-tailed)
Left to right parietal connectivity	0.00	0.37	0.11	0.02	*p* = 0.987
Left parieto-frontal connectivity	−0.01	0.22	0.07	−0.09	*p* = 0.923
Right to left parietal connectivity	0.14	0.50	0.15	0.96	*p* = 0.360
Right parieto-frontal connectivity	0.20	0.26	0.08	2.53	*p* = 0.030*
Left fronto-parietal connectivity	−0.05	0.37	0.11	−0.45	*p* = 0.665
Left to right frontal connectivity	−0.22	0.56	0.17	−1.31	*p* = 0.219
Right fronto-parietal connectivity	−0.08	0.51	0.15	−0.54	*p* = 0.598
Right to left frontal connectivity	0.08	0.15	0.05	1.78	*p* = 0.106

^*^Difference does not survive Bonferroni correction for multiple comparisons

**Fig. 5:**
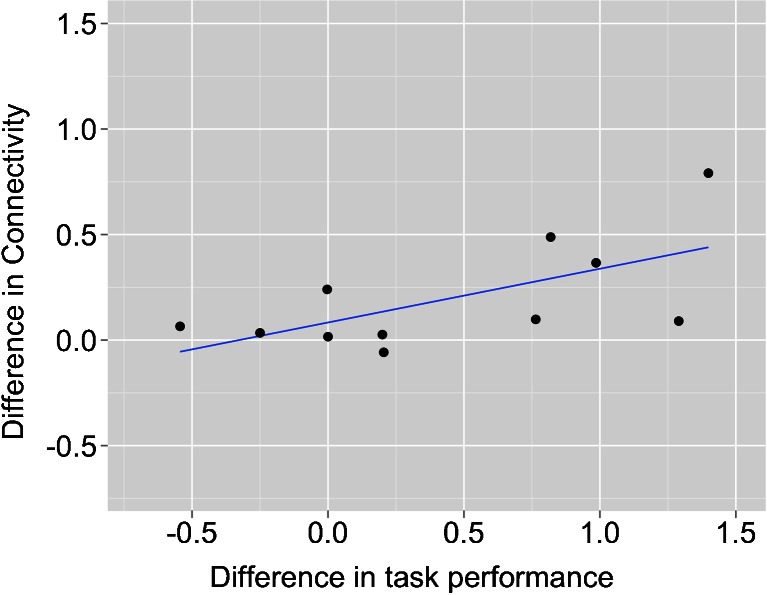
Significant positive correlation between the effect of green tea on task performance and SPL→MFG connectivity (green tea minus control substance; *r* = 0.64, *p* < 0.05). That is, the stronger the increase in SPL→MFG connectivity induced by green tea, the higher the improvement in the task performance compared with the control drink

Importantly, we found a significant positive correlation between the effect of green tea on task performance and right SPL → right MFG connectivity (*r* = 0.637, *p* = 0.035; Fig. 5).

## Discussion

In the present study we investigated the neural mechanisms underlying the putative beneficial impact on green tea extract on cognitive functioning. In particular, we explored by applying DCM to fMRI data whether green tea extract altered the WM-induced modulation of interregional effective connectivity between the parietal and frontal cortex. The main findings are that green tea extract increased the WM-induced modulation of connectivity from the right superior parietal lobule to the middle frontal gyrus. Furthermore, this effect of green tea on parieto-frontal connectivity positively correlated with its effect on task performance, suggesting a neural mechanism for the positive effect of green tea consumption on cognitive functioning at the system network level. Our finding of increased parieto-frontal coupling during WM processing induced by green tea might also explain the recently reported increase in prefrontal brain activity after green tea administration (Borgwardt et al. 2012). Thus, these studies together indicate that green tea extract might modulate WM processing by increasing prefrontal brain activity as a result of enhanced bottom-up connectivity from the parietal cortex.

Comparing competing models against the same data, we found that the family of models considering a bidirectional WM-induced modulation of connectivity between the parietal and frontal cortex had fitted the data of all participants best irrespective of treatments. This result supports previous functional connectivity studies emphasizing the importance of fronto-parietal connections for WM (Owen et al. 2005; Rottschy et al. 2012). The N-back task requires different cognitive processes including a continuous encoding of incoming visual letters and rule updating. Connections from the parietal to the frontal cortex (bottom-up) may contribute to the encoding of incoming stimuli (Ma et al. 2011), while the connections from the frontal to the parietal cortex (top–down) likely mediate the updating of rules (Gazzaley et al. 2004; Sauseng et al. 2005). Under this perspective, we may speculate that our result of enhanced parieto-frontal connectivity induced by green tea intake may indicate an improvement in stimuli encoding during the N-back task.

### Plasticity-dependent mechanism underlying the effect of green tea on cognitive functioning

Green tea mainly consists of polyphenols, particularly catechins such as (−)-epigallocatechin gallate (EGCG), caffeine, and theanine, as well a lot of additional ingredients. In the following, we show that these different substances share an overlap in activity of at least one biochemical pathway, the *N*-methyl-d-aspartate receptor (NMDAR) pathway, suggesting a plasticity-dependent mechanism that may link the cognitive effects of green tea from the micro- to the macro-level.

Studies in rodents support the idea of improved WM after green tea administration via catechin-induced promotion of antioxidative activity (Kaur et al. 2008). In accordance, previous studies proposed that EGCG mediates its protective effect on cognitive functioning through antioxidant and iron-chelating properties and modulation of cell-signaling and cell survival pathways (Mandel and Youdim 2004; Weinreb et al. 2004). In other words, EGCG appears to reduce oxidative stress (OS)-induced neurotoxicity as expressed by the generation of reactive oxygen species (ROS) generation. ROS generation is critically mediated by NMDAR-dependent flow of Ca^2+^ ions into neurons (Lafon-Cazal et al. 1993; Schanne et al. 1979). Treatment with green tea catechins as potent natural antioxidants completely normalized the response to activation of NMDAR by bath application of NMDA in the mouse brain, suggesting the involvement of OS in abnormal NMDAR-induced plasticity (Chepkova et al. 2013). Furthermore, EGCG promotes neural plasticity in the mouse hippocampus (Xie et al. 2008) and a facilitation of Ca^2+^-dependent glutamate release in rats (Chou et al. 2007). In Alzheimer disease, oligomeric Aβ attenuates NMDAR-mediated Ca^2+^ influx associated with an increase in ROS production (He et al. 2011). Furthermore, amyloid protein impairs synaptic plasticity by modulating an NMDA-type glutamate receptor-dependent signaling pathway (Shankar et al. 2007; Snyder et al. 2005). Specifically, amyloid-b (Ab) protein dimers isolated directly from Alzheimer’s brains disrupt synaptic plasticity and memory via inhibition of long-term potentiation and an enhancement of long-term depression (Shankar et al. 2008), both of which are critically mediated by NMDARs (Bliss and Collingridge 1993; Malenka and Nicoll 1993; Paoletti et al. 2013). Remarkably, EGCG decreases Aβ levels and plaques in mice, reduced Aβ mediated cognitive impairment and modulates tau pathology in Alzheimer transgenic mice (Lee et al. 2009; Rezai-Zadeh et al. 2005, 2008), as well as prevents Aβ-induced mitochondrial dysfunction, impairment of NMDA Ca^2+^ influx and ROS production (He et al. 2011). In addition to tea catechins, theanine, which is an amino acid uniquely found in tea leaf, may also possess neuroprotective effect (Nathan et al. 2006), probably by its antagonistic effect on ionotropic glutamate receptor subtypes, such as NMDARs (Kakuda 2011). Moreover, the beneficial effects of caffeine on stress-induced memory disturbance are mimicked by antagonists of adenosine A2a receptors, likely mediated by its ability to control glutamatergic transmission, especially NMDAR-dependent plasticity (Cunha and Agostinho 2010). Taken together, these studies suggest that green tea extract or its ingredients counteracts the OS-induced impairments in cognitive functioning via its effect on NMDAR-dependent synaptic plasticity.

In this study, we examined, at the network connectivity level, whether green tea intake altered the short-term plasticity of interregional connections between the frontal and the parietal cortex during WM processing by using DCM. DCM is a generic Bayesian system identification technique that allows inferring on NMDA-dependent synaptic plasticity by computing the dynamics of interacting neural macro-systems (Friston et al. 2003; Stephan et al. 2006, 2009a). Previous studies demonstrated the sensitivity of DCM for NMDAR stimulation (Moran et al. 2011) and that blocking of the NMDAR leaded to altered synaptic plasticity of the bottom-up connectivity from left primary auditory cortex to superior temporal gyrus during an auditory oddball task (Schmidt et al. 2013a). Thus, we propose that our result of an enhanced parieto-frontal connectivity during WM processing induced by green tea intake might reflect a green tea-induced modulation of NMDAR-dependent synaptic plasticity, suggesting a mechanism at the network level for the cognitive effect of green tea consumption.

### Limitations

There are some limitations to be considered in the present study. In contrast to the imaging results, we observed no significant effect of green tea consumption on task performances. However, we found a strong trend toward improved performance, suggesting that our study sample was too small to achieve differences on behavioural parameters. This suits with evidence that fMRI data on small subject numbers are relatively robust (Friston et al. 1999), while behavioral indexes are typically underpowered and could be confounded by many personal attributes that cannot be clearly assigned to the cognition required for adequate task performance (Wilkinson and Halligan 2004). A further caveat is that there is a difference between using a soft drink containing green tea and a pure green tea extract. Oral ingestion of pure green tea extract would have avoided any cross effects or effects of other components as caffeine that may be involved in the positive effect of green tea extract on cognitive performance.

## Conclusions

The present study shows that green tea extract enhances functional connectivity from the parietal to the frontal cortex during WM processing in healthy controls. Interestingly, this effect on effective connectivity was related to the green tea induced improvement in cognitive performance. Our findings provide first insights into the neural effect of green tea on WM processing at the neural network level, suggesting a mechanism on short-term plasticity of interregional brain connections. Our findings further suggest that the assessment of effective connectivity among frontal and parietal brain regions during working memory processing may provide a promising tool to assess the efficacy of green tea or other compounds for the treatment of cognitive impairments in psychiatric disorders such as dementia.
